# Efficacy and safety of Qigong Baduanjin exercise in the treatment of depression with insomnia: A randomized controlled study protocol

**DOI:** 10.1097/MD.0000000000027764

**Published:** 2021-11-24

**Authors:** Jing Fan, Fangmin Qian, Qingqing Wang, Bihua Chen, Linchuang Wang

**Affiliations:** a The Longhua Street Community Health Center, Xuhui, Shanghai, China; b The Shanghai Ruijin Rehabilitation Hospital, Shanghai, China.

**Keywords:** Baduanjin exercise, depression, insomnia, protocol, randomized controlled trial

## Abstract

**Background:**

Depression is a common mental illness often associated with insomnia. Baduanjin exercise has been found to improve depressive symptoms and has also been found to have good effects on insomnia. However, there are no rigorous clinical studies to evaluate the effects of Baduanjin exercise on depressed patients with insomnia, so this randomized controlled trial will evaluate the efficacy of Qigong Baduanjin exercise in treating depression with insomnia.

**Methods:**

This is a prospective randomized controlled trial to investigate the clinical efficacy of Baduanjin exercise in the treatment of depression with insomnia. The included patients will be randomly divided into a treatment group and control group. The treatment group will be treated with Baduanjin exercise and the control group will be treated with oral mirtazapine. After 8 weeks of continuous treatment, they will be followed up for 3 months. Observed indexes included Pittsburgh sleep quality index, Hamilton expression Rating Scale score, and adverse reactions. Finally, the data are statistically analyzed by SPSS 20.0 software.

**Discussion:**

This study will evaluate the efficacy and safety of Baduanjin exercise in the treatment of depression with insomnia, and the results of this study will provide a clinical basis for the treatment of depression with insomnia.

**Trial registration:** OSF Registration number: DOI 10.17605/OSF.IO/KC48H

## 1 Introduction

Major depressive disorder is a chronic psychiatric disorder characterized by significant and persistent depression. It is one of the most common mental disorders.^[1]^ Clinical manifestations include persistent depressed mood, slowed thinking, and reduced energy, while some patients may experience loss of appetite, low self-esteem and, in some cases, suicidal tendencies.^[2–4]^ Some studies have shown that the prevalence of major depression in China is 3.02%,^[5]^ while the prevalence of depression in China reaches 7.3% in 2020 as people's life stresses increase with the development of the times,^[6]^ while among adolescents, 24.6% are detected to have depression.^[7]^ Most patients with depression are accompanied by sleep disorders, with 1 survey showing that 90% of patients diagnosed with depression have sleep disorders.^[8]^ On the other hand, insomnia is a potential risk factor for depression, which can aggravate the condition and lead to prolonged depression, thus seriously affecting the patient's life and work, so effective treatment can better alleviate the patient's condition and reduce the huge financial burden on the family and society.

At present, anti-insomnia drugs are often used to treat depression with insomnia, but long-term oral western medicine will produce drug resistance, and the increasing drug dose and adverse reactions of drugs will affect the health of patients. Baduanjin exercise is a traditional Chinese Qigong sport, which emphasizes the integration of body and mind. As a low-energy exercise, Baduanjin exercise can not only enhance the physique, but also regulate people's mood, so that people can reduce pressure and relax in the process of exercising. It has the effect of prevention and adjuvant treatment of physical and mental diseases.^[9,10]^ Although it has been suggested that Baduanjin exercise can improve depression and sleep quality in patients,^[11]^ there are no rigorous randomized controlled studies to evaluate the clinical efficacy of Baduanjin exercise in the treatment of depression combined with insomnia, and there is a lack of long-term follow-up observations. Therefore, this study intends to evaluate the efficacy and safety of Baduanjin exercise in the treatment of depression with insomnia through a prospective randomized controlled trial.

## 2 Materials and methods

### 2.1 Study design

**Figure 1:**
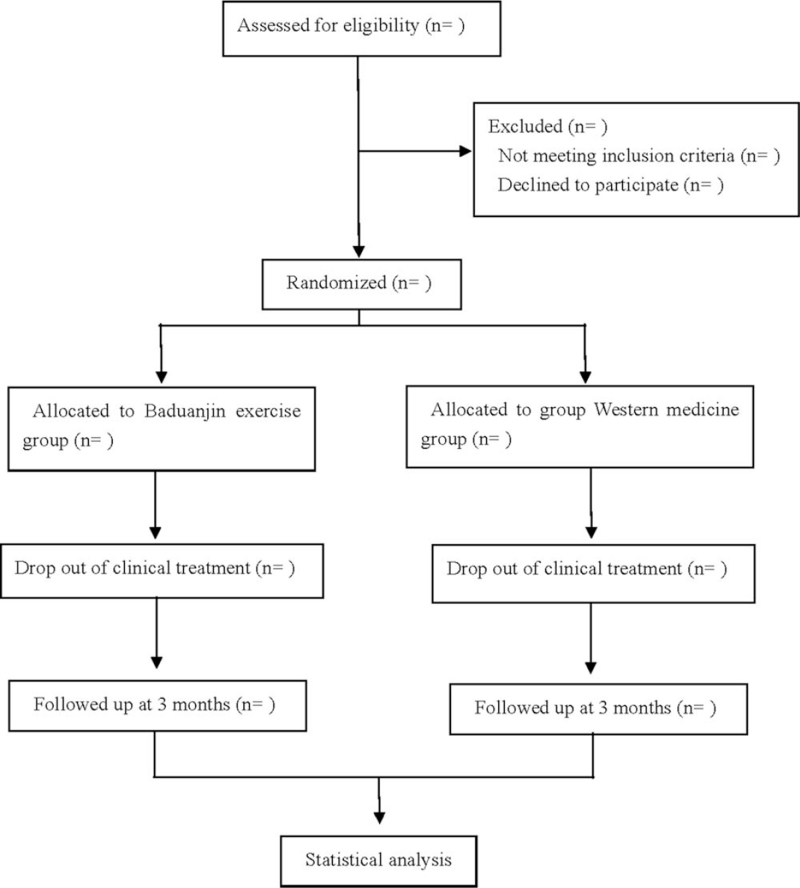
Flow diagram.

This is a prospective randomized controlled study to study the efficacy and safety of Baduanjin exercise in the treatment of depression with insomnia. The patients will be randomly divided into a treatment group and control group. The treatment group will take Baduanjin exercise, and the control group will take mirtazapine tablets orally. After 8 weeks of continuous treatment, they will be followed up for 3 months. The flow diagram is shown in Fig. 1. This research scheme follows the latest Consolidated Standards of reporting trials (2017) and standard protocol items: recommendations for interactive trials 2013 statement.

### 2.2 Ethics and registration

This research protocol complies with the declaration of Helsinki and ethical guidelines for clinical research, and has been registered in the open science framework (Registration Number: DOI 10.17605/OSF.IO/KC48H) with the approval of the Committee of clinical research ethics of our hospital. Before the start of the study, we will inform patients of the methods and potential risks of the study. After obtaining their written informed consent, they will be randomly divided into groups. During the study, they can freely choose whether to continue the trial at any time.

### 2.3 Patients

#### 2.3.1 Basis of diagnosis

Refer to the Chinese Classification and Diagnostic Criteria for Mental Disorders, 3rd Edition (CCMD-3)^[12]^ for the diagnostic criteria of depression and insomnia.

#### 2.3.2 Inclusion criteria

(1) Meet the diagnostic criteria for depression and insomnia.
(2) Those with a Hamilton Depression Rating Scale score ≥24.
(3) Age ≥18 and ≤60 years.
(4) Not taking antidepressant or sleep-aid medication (including traditional Chinese medicine and western medicines) within the last 2 weeks.
(5) Patient signed an informed consent form.

#### 2.3.3 Exclusion criteria

(1) Patients with a psychiatric disorder other than depression.
(2) Patients with insomnia due to other diseases or medications.
(3) Patients with alcoholism, psychoactive substances, drug abusers, and those who are dependent.
(4) Women who are pregnant, preparing for pregnancy, or breastfeeding.
(5) Those who have participated or are participating in other clinical trials within the last 1 month.

#### 2.3.4 Discontinuation criteria and treatment

(1) Those who experience serious adverse events or other complications, etc, and who, in the judgment of the investigator, should be discontinued from the trial and given appropriate life-saving treatment.
(2) Subjects who have poor compliance and are unable to complete the entire set of Baduanjin exercise movements, which have an impact on the study results.
(3) Subjects who, for whatever reason, are unwilling or unable to continue with the clinical trial and request the investigator to withdraw from the trial and discontinue the trial.

For discontinued cases or lost cases, the investigator should complete the last test possible for analysis of their efficacy and safety. For all discontinued cases, the reason for the discontinuation should be entered on the case report form.

### 2.4 Sample size

Sample size estimates are based on the mean and standard deviation of the Pittsburgh sleep quality index^[13]^ scores of patients after 8 weeks of treatment, referencing the pre-test results of 11.24 ± 5.03 for the treatment group and 14.27 ± 5.31 for the control group, set at α = 0.025, one-sided test, β = 0.20. Calculated by PASS 15.0 software to require 47 participants per group. With an estimated drop-out rate of 10%, 53 patients will be included in each group.

### 2.5 Recruitment strategy and distribution plan

Participants will be screened among patients attending outpatient clinics and recruitment information will be disseminated through the distribution of brochures and posted on the Internet and WeChat platforms to widen the scope of screening. Participants who meet the inclusion criteria will be fully informed of the purpose and protocol of the study and informed consent signed by the participants will be obtained.

The study will randomly assign patients who meet the criteria for either the treatment group (Baduanjin exercise group) or the control group (Western medicine group) in a 1:1 ratio through a central network-based randomization tool. The random numbers are generated by a statistician using a computer software program and these random numbers are placed in sealed opaque envelopes which are handed to the participants by the study coordinator. Due to the specific nature of the intervention, the patient and the operating physician are knowledgeable about the assigned outcome. However, the assessors of the study results and the statisticians who counted and analyzed the data are unaware of the allocation.

### 2.6 Interventions

Patients in both groups will be given health guidance and psychological counseling and maintained their current lifestyle. The treatment group will receive regular Baduanjin exercise. The Baduanjin exercise used in this study is the Baduanjin exercise of Fitness Qigong developed by the General Administration of Sports of China, which consists of 8 main movements. A medical practitioner who has been practicing Baduanjin exercise for 10 years will explain the key points of Baduanjin exercise and give step-by-step instructions to ensure that each patient learns it completely and is issued with a DVD tutorial. Patients will practice at home once a day, 5 times a week, for 30 minutes each time for a total of 8 weeks. The control group will be given oral mirtazapine tablets (N.V. Organon, Netherlands, H20090160) at 15 mg once a day at bedtime, 5 times a week for 8 weeks.

Patients will record in the case report form details of the time taken or practiced, and any discomfort that occurs during the study.

### 2.7 Outcomes

(1) Pittsburgh sleep quality index^[13]^: including sleep quality, time to sleep, sleep duration, sleep efficiency, hypnotic medication, sleep disturbance, and daytime functioning. After scoring respectively, the total score is 21. The higher the score, the worse the sleep quality.
(2) Hamilton Depression Rating Scale^[14]^: it includes 24 items. The higher the score, the heavier the symptoms.
(3) Incidence of adverse reactions: including treatment-related discomfort experienced by patients during the study period.

Two research assistants who are unclear about the outcome of the subgroups will score the 2 groups of patients according to outcome indicators before treatment, at the end of treatment, and at month 1 and month 3 of the end of treatment, through outpatient visits.

### 2.8 Data collection and management

During the study, personal information about potential and enrolled participants will be collected, shared, and kept in a separate repository to protect confidentiality before, during, and after the trial. Any modifications or changes to the protocol will be reapproved through a formal hospital ethics committee process. An independent clinical research assistant will review the progress of the study on a regular basis. Personnel outside of this research team do not have access to relevant information.

### 2.9 Statistical analyses

Independent statisticians with unclear results for random assignment will be responsible for the statistics and analysis of the data, and the data collected will be statistically analyzed by SPSS 20.0 software. The chi-square test will be used for count data; mean ± standard deviation ($\bar{x}\pm \text{S}$) will be used for measurement data, independent samples *t* test for normal distribution and Mann–Whitney *U*-test for skewed distribution, and differences will be considered statistically significant at *P* < .05.

## 3 Discussion

The development of depression is related to a variety of factors, including genetics, social relationships, and neurobiology.^[15,16]^ Insomnia is not only a symptom of depression, but also affects a person's mood and behavior, increasing the risk of developing depression,^[17]^ and some studies have shown^[18]^ that insomnia patients are far more likely to develop depression than the general population. Therefore, it has been suggested that treating depression and insomnia together results in better overall symptom improvement for patients than treating depression alone.^[19]^

Baduanjin exercise is a traditional Chinese exercise that can improve the function of the internal organs of the body by regulating the flow of Qi and blood through the meridians to achieve the effect of fitness and dispel diseases.^[20]^ At present, Baduanjin exercise has been widely used in the treatment of diabetes mellitus,^[21]^ chronic obstructive pulmonary disease,^[22]^ and heart failure,^[23]^ and has achieved good results. Clinical studies have found that Baduanjin exercise improves the symptoms of depressed patients by regulating the expression of lncRNA, mRNA, and circRNA,^[24]^ and are also effective in improving sleep quality.^[25]^ There are no rigorous clinical studies to verify whether the same efficacy of Baduanjin exercise exists in patients with both depression and insomnia. Therefore, this study evaluates the efficacy and safety of Baduanjin exercise in the treatment of depression with insomnia through a rigorous randomized controlled protocol to provide a clinical basis for its dissemination.

Due to the small sample size and the regionalization of the included population, the results may be affected to some extent; Although this study was followed up for 3 months, it may still be impossible to objectively evaluate the long-term effect of Baduanjin exercise on patients with depression and insomnia. We look forward to more metacenter and large sample randomized controlled studies to verify our conclusions.

## Author contributions

**Conceptualization:** Jing Fan, Fangmin Qian.

**Data curation:** Jing Fan, Qingqing Wang.

**Formal analysis:** Fangmin Qian, Qingqing Wang, Jing Fan.

**Funding acquisition:** Linchuang Wang.

**Software:** Fangmin Qian, Linchuang Wang.

**Supervision:** Qingqing Wang, Linchuang Wang.

**Writing – original draft:** Fangmin Qian, Bihua Chen.

**Writing – review & editing:** Qingqing Wang, Bihua Chen.
